# Diabetic Retinopathy Screening Among at Risk Populations: Protocol for Distributional Cost-Effectiveness Analysis

**DOI:** 10.2196/60488

**Published:** 2025-04-30

**Authors:** Aleksandra Stanimirovic, Troy Francis, Sonia Meerai, Suja Mathew, Sarah Ibrahim, James M Bowen, Aleksandra PIkula, Valeria Rac

**Affiliations:** 1 Toronto General Hospital Research Institute Ted Rogers Centre for Heart Research at Peter Munk Cardiac Centre University Health Network Toronto, ON Canada; 2 Institute of Health Policy, Management and Evaluation University of Toronto Dalla Lana School of Public Health Toronto, ON Canada; 3 Diabetes Action Canada CIHR SPOR Network Toronto, ON Canada; 4 Toronto Health Economics and Technology Assessment Collaborative Toronto General Hospital Research Institute University Health Network Toronto, ON Canada; 5 Program for Health System and Technology Evaluation Toronto General Hospital Research Institute University Health Network Toronto, ON Canada; 6 Faculty of Education and Health School of Social Work Laurentian University Sudbury, ON Canada; 7 The Jay and Sari Sonshine Centre for Stroke Prevention & Cerebrovascular Brain Health Toronto Western Hospital University Health Network Toronto, ON Canada; 8 Centre for Advancing Collaborative Healthcare & Education University of Toronto Toronto, ON Canada; 9 Department of Health Research Methods, Evidence & Impact, Faculty of Health Sciences McMaster University Hamilton, ON Canada; 10 Department of Medicine (Neurology), Temerty Faculty of Medicine University of Toronto Toronto, ON Canada; 11 Krembil Brain Institute University Health Network Toronto, ON Canada

**Keywords:** diabetic retinopathy, equity of care, distributional cost-effectiveness analysis, health equity, health care disparities, intersectionality, telehealth, telemedicine, retinopathy screening

## Abstract

**Background:**

Diabetic retinopathy (DR) remains the primary vision complication of diabetes and the leading cause of blindness among adults, with up to 30% prevalence among low-income populations. Tele-retina is a cost-effective screening alternative to vision loss prevention, yet there is an adverse association between screening and income. Intersectionality theory notes that barriers to achieving health equity result from the intersection of personal and social characteristics. Experiences at this intersection are influenced by interpersonal and structural systems of oppression. Studies have found that tele-retina is the preferred strategy over standard of care screening for at-risk populations. No study has assessed the economic equity impact of DR screening using a theoretical foundation.

**Objective:**

This study aims to address shortcomings related to the utilization of intersectionality theory in the economic evaluation of DR screening. We propose conducting a distributional cost-effectiveness analysis (DCEA) of the tele-retina program.

**Methods:**

The study will be undertaken using a deductive theoretical drive sequential multimethod approach, consisting of two studies: (1) a modified Delphi study and (2) DCEA. The Delphi panel (patient partners, field experts, and decision makers; N=35-50) will select the social constructs (eg, age, gender) for at-risk populations and potential trade-offs between health maximization and equity. The research will be guided by a social theory framework (intersectionality theory) to understand the impact of social constructs on economic outcomes. Social constructs that are selected by the Delphi panel will be integrated into the validated tele-retina cost-effectiveness analysis model, which will serve as a case study for DCEA.

**Results:**

We have submitted the research ethics board application to the University Health Network Research Ethics Board and are expecting to begin recruitment for the Delphi study in Spring 2025. We anticipate beginning work on the model in the summer of 2025 and completing it by early 2026.

**Conclusions:**

The Delphi study will provide an understanding of which social factors are deemed necessary by the stakeholders for guiding the inequity in care access. Study results will offer information related to the net health benefit of the intervention and the health equity impact of the tele-retina program, hence providing a more comprehensive valuation of the tele-retina program, which is informative to policy makers and governments whose goal is to mitigate the drivers of health inequities. We anticipate that each of these drivers will raise important questions regarding the implications for decision-making that may have not yet been addressed by Canadian health technology assessment bodies, such as the Canada Drug Agency. This is the first Canadian study to (1) have social constructs for DCEA selected by the Delphi panel, (2) mainstream how health equity framework and social constructs are used in economic assessment, (3) improve DR screening programs by using health equity lens, and (4) scale and adopt “de-novo” integration of social constructs in economic models for program evaluation.

**International Registered Report Identifier (IRRID):**

PRR1-10.2196/60488

## Introduction

### Social and Economic Impacts of Diabetes

Every 3 minutes, someone in Canada is diagnosed with diabetes, while 11 million are living with diabetes or prediabetes [1]. About 6.1% of Canadian adults aged 20-79 years have prediabetes, putting them at high risk of developing type 2 diabetes [1]. The prevalence of diabetes is expected to increase to 5 million (12.1%) by 2025 [1]. Ontario has more people living with diabetes than anywhere else in the country. Rates of type 1 and type 2 diabetes have increased by 42% since 2009 [1]. There are now an estimated 4,424,000 people with diabetes or prediabetes in Ontario [1]. Diabetes Canada states that the costs of treating diabetes have surpassed CAN $30 billion [2] (a currency exchange rate of US $0.70). Goeree et al [3] have estimated that the excess attributable cost per incident case per person of diabetes in Ontario is CAN ~$2930 in the first year after diagnosis and CAN $1240 in the following years [3]. In Canada, diabetes disproportionately impacts First Nations and Métis people, and people of African, East Asian, and South Asian ethnic backgrounds, who experience higher rates of type 2 diabetes compared to the general population [4]. Inequities in the social determinants of health (eg, income, education, and housing), resulting from the impacts of systemic racism, intergenerational trauma, and colonization, are associated with higher rates of type 2 and gestational diabetes in priority populations [4].

### Equity in Health Service Delivery and Intersectionality Framework

**Figure 1:**
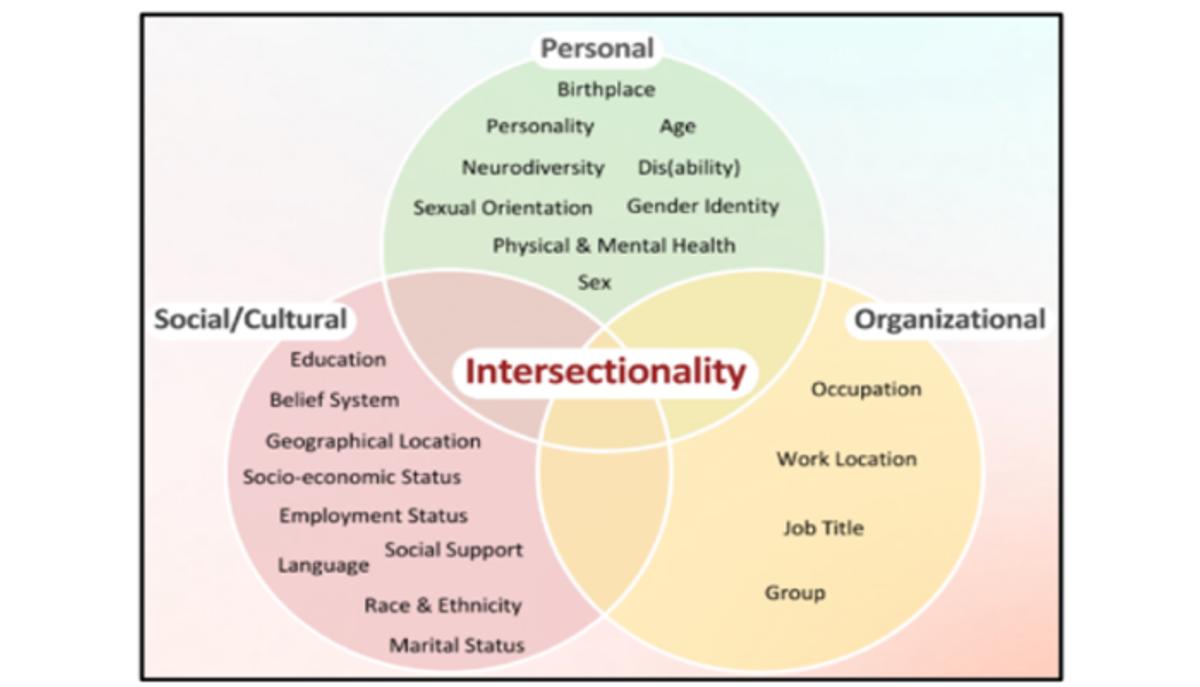
Intersectionality or synergies of oppression. SDH: social determinants of health.

Health equity is created when individuals have a fair opportunity to reach their fullest health potential. It is directly correlated with the distribution of resources within any setting [5]. Low socioeconomic groups with multiple health conditions and limited access to resources have decreased access to achieving health equity [5]. Intersectionality (Figure 1) is a theoretical framework in which consideration of heterogeneity across different intersections of social positions is critical to the conceptualization of health and social experiences [6]. The framework was published by Crenshaw [7] and developed within Black feminist theory to better explicate the situation of Black women in the United States [8]. It encompasses a wide range of intersections of ethnoracial groups, gender, socioeconomic status, sexual orientation, and other social identities and positions [7]. The framework notes that social positions exist on a hierarchy of social power and are not independent [7] but rather that they shape human experience jointly. As social positions intersect at the individual level (eg, income, age, and rurality), experiences at those intersections are influenced by larger interpersonal and structural systems of oppression, such as racism and sexism [9]. The synergies of oppression are created through the intersections of three domains: (1) socioeconomic status (ie, income), (2) identity-isms (eg, race), and (3) geography (segregation). Intersectionality in this conceptual framing (Figure 1) is an intersectional categorical axis where social determinants of health, systems of oppression, and environmental factors are integrated and three categories or framings within the context of the synergy of oppressions, specifically the outcomes of increased marginalization through oppressions and intersections of social determinants of health. This synergy is further compounded with access to health equity [7-9].

### Social and Economic Impacts of Diabetic Retinopathy and Screening

Diabetic retinopathy (DR) is the primary vision complication caused by diabetes [10] and is the leading cause of new cases of blindness in adults aged 20-65 years [11], yet it is often asymptomatic in the initial stages [12]. The prevalence of DR in Canada ranges from 20% to 30% [12]. In 2016, more than a million Ontarians were affected by DR [6]. The prevalence of vision loss in Canada is expected to increase by nearly 30% in the next decade [13]. Lower income and type 2 diabetes are associated with increased odds of visual impairment [14]. In 2007, the cost of vision loss in Canada was estimated at CAN $15.8 billion and is projected to grow to CAN $30.3 billion by 2032 [12]. In addition to these costs, the Canadian National Institute for Blindness estimated the cost of associated complications of vision loss: falls CAN $25.8 million, depression CAN $175.2 million, hip fractures CAN $101.7 million, and nursing home admissions CAN $713.6 million [12]. Early detection ensures that treatment or intervention can decrease the incidence of vision impairment or blindness. Evidence notes that one-third of adult patients with diabetes did not receive an eye examination for DR within 2 years [6], and more specifically, 25.3% of people with diabetes over 60 years had not seen an eye care provider in the last year [12]. Tele-retina is one of the DR screening modalities that can be used to bring the testing to community settings. It is focused on reducing eye care disparities that lead to avoidable vision loss. Tele-retina is a branch of telemedicine that delivers eye care remotely. Retinal images and data are collected and transferred via telecommunication technology to eye specialists [15]. Previous research has noted that it is a more cost-effective alternative than the standard screening in detecting DR among lower-income individuals with limited access to eye care [15]. Specifically, the cost per case correctly detected was CAN $281.10 with tele-retina and CAN $982 with the standard of care (SOC) screening (note, the standard of care screening for DR entails walking into an optometrist or ophthalmologist office to get the eyes checked), and the cost per case correctly diagnosed was CAN $82.21 and CAN $314.14, respectively. For both pilot and pan-Ontarian sensitivity analyses, tele-retina remained the dominant strategy (ICER <0). Unfortunately, screening rates among at-risk populations remain below 60% [12,15].

For DR screening, we know that socioeconomic status is the primary driver of risk for DR screening and that systemic societal barriers, for example, ageism, racism, and sexism [7,8], have had a detrimental impact on eye health and vision. Lack of education or information, social role and identity, competing priorities (getting time off work or study to attend appointments), language barriers, and lack of family and clinical support [9,10] are noted by patients as barriers associated with access to DR screening.

Differential access to retinopathy screening was evident when we evaluated the designed tele-retina model of DR screening. We found that only 50% of low-income individuals living with diabetes with limited access to vision care would get screened through SOC screening (walking into an optometrist or ophthalmologist office to get their eyes checked, which may be even more challenging in remote and rural areas), whereas 80% of the same population would get screened with the tele-retina program [11]. Screening percentage was the major driver of tele-retina’s economic dominance. This further suggests the need to elucidate social factors associated with the inequity of screening, as ignorance of these remains a major factor in perpetuating lack of access to equitable care.

### Overall Objective

In collaboration with patient partners, the primary objective of this project is to assess the equity impacts of tele-retina screening in populations that experience systemic social and economic disadvantages with limited access to conventional eye care.

More specifically, we will:

- Create an inventory of social constructs that could be used in subsequent (economic) analysis when tackling inequity in the health care setting
- Apply a novel methodological theoretically guided framework for undertaking a distributional cost-effectiveness analysis (DCEA) to combine the objectives of maximizing health and minimizing unfair variation in health when evaluating population health interventions using a case study of DR screening

Understanding the equity impact of the tele-retina program will contribute to the creation of socially and culturally acceptable economic milieu in line with the multicultural Canadian context, with the ultimate purpose of improving the screening rate for DR, augmenting the quality and access to DR care, and decreasing the incidence of severe vision loss ultimately leading to blindness.

## Methods

### Study Design

**Figure 2:**
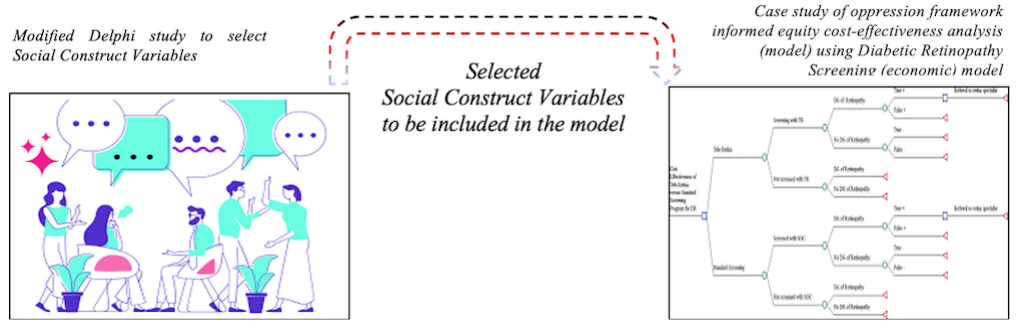
Illustration of the study design.

This study will build on our current intersectionality work for DR screening access among those who identify as women of low socioeconomic status [16]. Meanwhile, combining the advantages of multiple designs and enabling a comprehensive understanding of social complexities related to the implementation context, we propose the deductive theoretical drive sequential multimethod approach. Multimethod design entails conducting two or more research methods rigorously and completely in one project, where the major research question drives the study, but the approach consists of 2 interrelated studies. The deductive theory-driven approach indicates that we begin with an (intersectionality) theory, develop a hypothesis from that theory, and then collect and analyze data to test our hypotheses. The sequential approach indicates quanàQUAN, encompassing two quantitative methods used sequentially, one of which is dominant (in capital letters) [17,18]. The two components of this study are (1) modified Delphi and (2) DCEA study. Once the first project (modified Delphi) is completed, we will use the results to provide details for the DCEA (Figure 2).

### Stage 1: Modified Delphi

In the modified Delphi, we are seeking the opinion of participants as to whether social constructs may dictate one’s health (seeking) behavior. The Delphi will guide us in developing the set of social construct variables to be integrated into the tele-retina screening economic model. We anticipate 35-50 participants (patient representatives: n=20-25; field experts, including in health technology assessment [HTA], and clinicians: n=5-10; decision and policy makers: n=10-15) who will be purposely selected based on experience, training, and specialized areas of knowledge (Table 1). Please note that there is no standard size of the Delphi panel members, and it varies from 10 to 1000 (typically between 10-100) in published studies. However, due to data management difficulties and logistic issues (rounds of the survey), a panel with a 3-digit sample size is unusual [1,2]. Generally, a double-digit number close to 30-50 is considered optimal in concluding rounds for a homogenous Delphi [3,4]. Appropriate size depends on the complexity of the problem, homogeneity (or heterogeneity) of the panel, and availability of the resources. Apart from panel members with knowledge, some studies recruit members from diverse academic and practice backgrounds or involve end users in the process [5]. Delphi participants are all members of the Diabetes Action Canada (DAC) Network. Potential participants, who will be invited via Network email to participate [19], will consist of patient partners, researchers, diabetes specialists, primary care practitioners, nurses, pharmacists, data specialists, and health policy experts committed to improving the lives of persons with diabetes. The team has successfully partnered and collaborated with DAC to plan, execute, and evaluate research projects to improve patient outcomes and experiences [20]. Before joining the Delphi panel, each participant will be approached by a qualitative researcher and study lead to sign a consent form.

**Table 1:** Modified Delphi general categories of expertise.

General categories of expertise	Experts, n
Patient representatives	20-25
Field experts, including in health technology assessment, and clinicians	5-10
Decision and policy makers	10-15

Below is a summary of the modified Delphi, as it is a well-established approach to answering a research question by identifying a consensus view across subject experts.

### Approach to Analysis

#### Overview

Social construct selection (income, education, area-level deprivation, ethnicity, area-level ethnic diversity, sex, and gender) is guided by the constructs’ baseline distribution and will be presented to the Delphi participants by patient partners and qualitative researchers. The baseline distribution informs on one’s physical, mental, and emotional health. It is shaped by one’s medical, social, and family history, and is continuously influenced by factors affecting everyday life factors [17,18,21]. Relevant population characteristics include dimensions of direct equity concern (eg, income, area-level deprivation, and ethnicity or area-level ethnic diversity) and characteristics that are necessary to estimate expected costs and effects and generate further equity concern (eg, sex and gender) [17,18,20,21]. We will undertake a 2-round process to assess the importance of each variable (social construct) on a 0-5 Likert scale (not at all important, low importance, neutral, moderately important, and very important) [22].

#### Area-Level Deprivation

The construct allows for rankings of neighborhoods by a socioeconomic disadvantage in a region of interest (eg, at the state or national level). It includes factors for the theoretical domains of income, education, employment, and housing quality.

#### Area-Level Ethnic Diversity

Based on the Statistics Canada census the diversity index is a measure of the racial and ethnic diversity of residents based on 7 major racial and ethnic or political groups (Asian American, Black, Latinx, Pacific Islander, mixed or other race, Native American, and White) identified by the census. The value of the area-level ethnic diversity ranges from 0 to 0.93. A diversity index of 0 means that every person in the area belongs to the same ethnic group. The highest possible index of 0.93 (based on the current census) corresponds to a perfect mixture of ethnic groups, with an equal proportion of each ethnic group in the region.

#### Round 1

At a workshop, information about variables will be presented to panelists, and subsequently, the panel will answer the Delphi questionnaire (Multimedia Appendix 1). Questionnaire responses will be entered into Microsoft Excel; frequencies will be calculated; and after Round 1, a summary report will be prepared. The report will be distributed to panel members. The need for further consideration of each social construct will be reviewed and confirmed through discussion at the meeting.

#### Round 2

The Delphi web-based questionnaire will be submitted 4 weeks following the workshop. The median score and range will be estimated for Likert scale questions per guidelines [22]. A proportion of 70% or higher viewing each domain and topic as “moderately important” versus “highly important” will be considered as an indication of the stability of participants’ consensus concerning face validity [22]. SAS 9.4 (SAS Institute) will be used to analyze the data.

### Case Study of Oppression Framework–Informed Equity Cost-Effectiveness Analysis

Once Delphi participants have selected the most relevant social constructs or variables to be included in the distributional cost-effectiveness model, we will rank them and select the 5 social constructs with the highest ranking to be considered for inclusion in the model.

**Figure 3:**
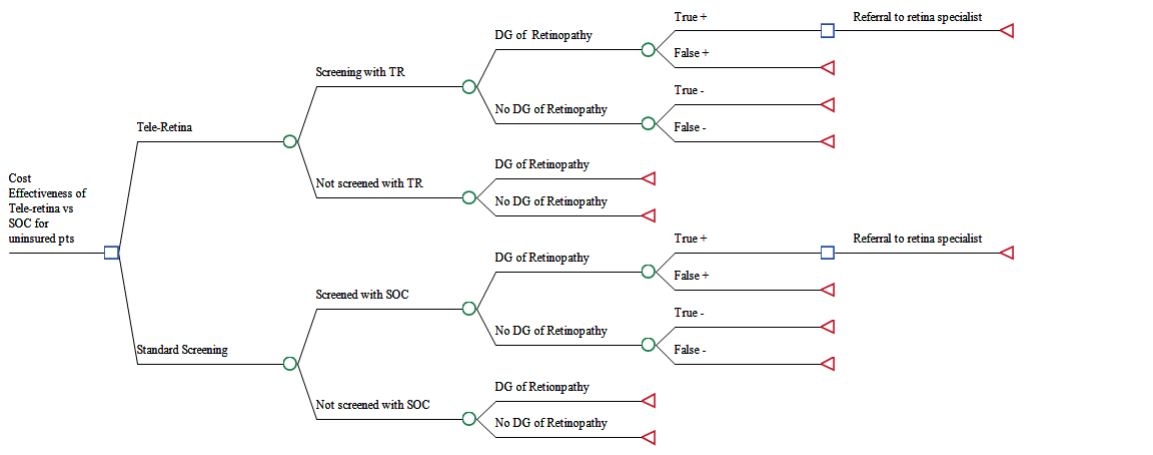
Decision tree of tele-retina versus standard of care screening (SOC) for diabetic retinopathy (DR). Decision tree illustrating the possible consequences, the chance of event outcomes, resource costs, and utility of using tele-retina (TR) versus in-person standard of care screening to measure diabetic retinopathy [15].

In the following, the modified Love-Koh et al [19] and Asaria et al [23,24] approaches (stages A and B as described in detail below), directed by a senior health economist, will evaluate which DR screening intervention has the best “social value” based on selected social constructs in the modified Delphi, as these are deemed important to the participants in health care resource allocation. We will investigate how these fit with the implicit value judgments inherent in the original cost-per-quality-adjusted life years model formulation of the tele-retina screening program. Note that quality-adjusted life years are a measure of disease burden, including both the quality and the quantity of life lived [25]. The case study is based on the Toronto tele-retina screening program offered in partnership with 7 primary care organizations with a population focus on those located in low-income urban and rural communities with a high prevalence of diabetes and low DR screening rates, with more details described in Figure 3 [15]. The Toronto tele-retina model was selected because it is a validated economic model but does not provide information on equity in the distribution of costs and effects. Please note that the case study population includes individuals residing in Ontario who are unable to obtain government insurance coverage (non–Ontario Health Insurance Plan [OHIP]–insured patients), including those residing illegally, those new to Canada awaiting coverage, and those so marginalized that they may struggle to obtain the identification and home address required to receive OHIP.

### Distributional Cost-Effectiveness Analysis

#### Stage A: Model Social Distributions of Health

##### Estimating the Baseline Health Distribution

We will describe the baseline distribution of health among the study population (length and health-related quality of life). Baseline distribution will need to include the full general population and not just the population receiving the intervention (tele-retina and SOC). This is important for 2 reasons. First, the general population is typically the relevant population for characterizing policy concerns with health inequality. Second, within a national, budget-constrained system, additional resources used by recipients of the intervention may displace activities that could have been provided to anyone within the full general population. This baseline distribution of health will describe variation in health among multiple different subgroups in the population as defined by relevant population characteristics, allowing for a correlation structure among these various characteristics. The relevant population characteristics include not only dimensions of direct equity concern (eg, income and ethnicity) but also characteristics necessary to estimate expected outcomes and costs that may generate further equity concern (eg, sex, gender). In consultation with the senior health economist, we will select the most appropriate health metrics ensuring that they are measured on an interpersonally comparable ratio scale suitable for use within a cost-effectiveness analysis [24] and their data availability. To obtain health metrics, we will consult with data from published literature based on individual studies, a systematic review to pool the data, published literature dealing with administrative data, and access to DR screening data from the Alliance for Healthier Communities, which includes data on uninsured patients.

##### Estimate the Distribution of Health Changes Due to the Interventions

To evaluate changes in the baseline health distribution that could be attributed to the use of alternative interventions, it is necessary to know how the outcomes and costs of the intervention differ between the relevant subgroups, and how the opportunity costs of any change in resource use differ by those same subgroups. After we estimate a baseline health distribution, we will model how this health distribution is affected by the tele-retina screening program and alternative ways of promoting increased uptake of the tele-retina screening. We do this by using an existing cost-effectiveness analysis Toronto tele-retina screening model [15] for the DR screening (Figure 3) that simulates the natural history of the disease and the impact of screening. The model compares in-person examination for DR screening (SOC) versus the tele-retina program [15]. The model has been conceptualized for patients residing in neighborhoods with limited access to ophthalmologists and optometrists at high risk for diagnosis of DR. The SOC is a fundus examination with pupil dilation performed by a primary eye care specialist (optometrist or ophthalmologist). Patients with positive results would be referred to a retina specialist, as coordinated by the primary care provider for comprehensive eye examination with angiography and optical coherence tomography. The model (Figure 3) illustrates pathways of care, possible consequences, chance of event outcomes, resource costs, and utility of using tele-retina versus in-person SOC to measure DR. The published tele-retina model ends with severe vision loss following screening alternatives. However, we have conceptualized and presented to the decision-making organization a complete model containing treatment effects. We will use the model with treatment effects for the case study.

We note that there are parameters in the model that can vary, including the following:

1. Disease prevalence, severity, mortality rate, and natural history: We assume in our case study that DR-specific parameters may be constant across our population subgroups (may need to confirm with expert)
2. Uptake of the intervention: The impact of DR screening uptake by subgroup is the key difference between the various implementations of the screening program’s success. We will estimate this parameter for each subgroup.
3. Direct costs associated with the intervention: We assume the direct costs related to treating a given stage of DR do not vary by subgroup (although the chance of incurring these costs and the screening-related costs by subgroup may vary under the different implementations of the screening program). This seems to be a plausible assumption without more detailed cost data at the subgroup level.
4. Opportunity costs from displaced activities: We assume that opportunity costs in the base case analysis are shared equally among all population subgroups; this assumption may be explored in sensitivity analyses.
5. Other-cause mortality: The mortality rates may vary by subgroup in the same way as discussed when deriving the baseline health distribution. In calculating these rates, we remove diabetic-specific mortality (assuming this is constant across subgroups) and apply this separately in the model.

##### Adjusting for Social Value Judgments About Fair and Unfair Sources of Inequality

The distributions of health estimated thus far would represent all variations in health in the population. We may perceive that some variation in health seems “fair,” or at least “not unfair,” perhaps because it is due to individual choice or unavoidable bad luck. Then, before measuring the level of inequality, we will first adjust the health distributions to only include health variation deemed “unfair.” We need to make social value judgments about whether health variation associated with each of the population characteristics is deemed fair. We will adjust our modeled health distributions for this value judgment by using the direct standardization method [26].

#### Stage B: Evaluating Social Distributions of Health

Once we have estimated the appropriate health distributions, we can characterize the distributions in terms of the twin policy goals of improving total health and reducing health inequality.

##### Comparing Interventions in Terms of Total Health and Unfair Health Inequality

We will use a specific index to compare the interventions in terms of total health and unfair health inequality. One useful piece of information for decision makers produced at this step of the analysis is the size of the health opportunity cost of choosing an intervention that reduces health inequality; this is simply the difference in total health between the intervention and a comparator. However, we can go further than that and provide information about the size of the reduction in health inequality in terms of the difference in one or more suitable inequality indices between the intervention and a comparator. The selection of appropriate inequality indices requires further value judgments about the nature of the inequality concern. There are a number of commonly used indices to measure inequality that can be broadly grouped into those measuring relative inequality (scale-invariant index) and those measuring absolute inequality (translation invariant). We can calculate the range of relative and absolute inequality measures for the quality-adjusted life expectancy distributions associated with our interventions. A higher value for each measure typically indicates a higher level of inequality between the healthiest and the least healthy. We will select the inequality measure in consultation with a senior health economist.

##### Ranking Interventions Using Dominance Rules Accounting for the Level of Inequality

To rank distributions based on mean health and the level of health inequality, we may use economic dominance rules provided by Atkinson [27] and Shorrocks. Dominance rules apply when mean health is higher and inequality is lower for almost any measure of inequality. Both rules are based on the Lorenz curve, which will be used to analyze relative inequality constructed for health distributions. We will do this by ordering the population from least healthy to most healthy and plotting the cumulative proportion of population health against the cumulative proportion of the population. Regarding Atkinson’s theorem tests for Lorenz dominance between distributions, this means that the Lorenz curves for the distributions do not cross, and the more equal distribution has at least as much mean health as the less equal distribution. In other words, a distribution is dominated if it has higher inequality and the same or lower amount of mean health. On these criteria, the standard screening strategy is dominated by the targeted reminder. Shorrocks’ theorem tests for generalized Lorenz dominance, wherein the Lorenz curve is multiplied by the mean health. We will consider that distribution is dominated if the generalized Lorenz curve [28] lies wholly below that of an alternative intervention. Under this criterion, the targeted and universal reminder strategies dominate the no-screening option. This leaves us to compare the universal reminder and targeted reminder strategies. However, if the generalized Lorenz curves for two distributions cross, we cannot use Shorrocks’ theorem to rank the distribution and will use Atkinson’s [27] theorem test for Lorenz dominance.

##### Analyzing Trade-Offs Between Total Health and Health Inequality Using Social Welfare Indices

**Figure 4:**
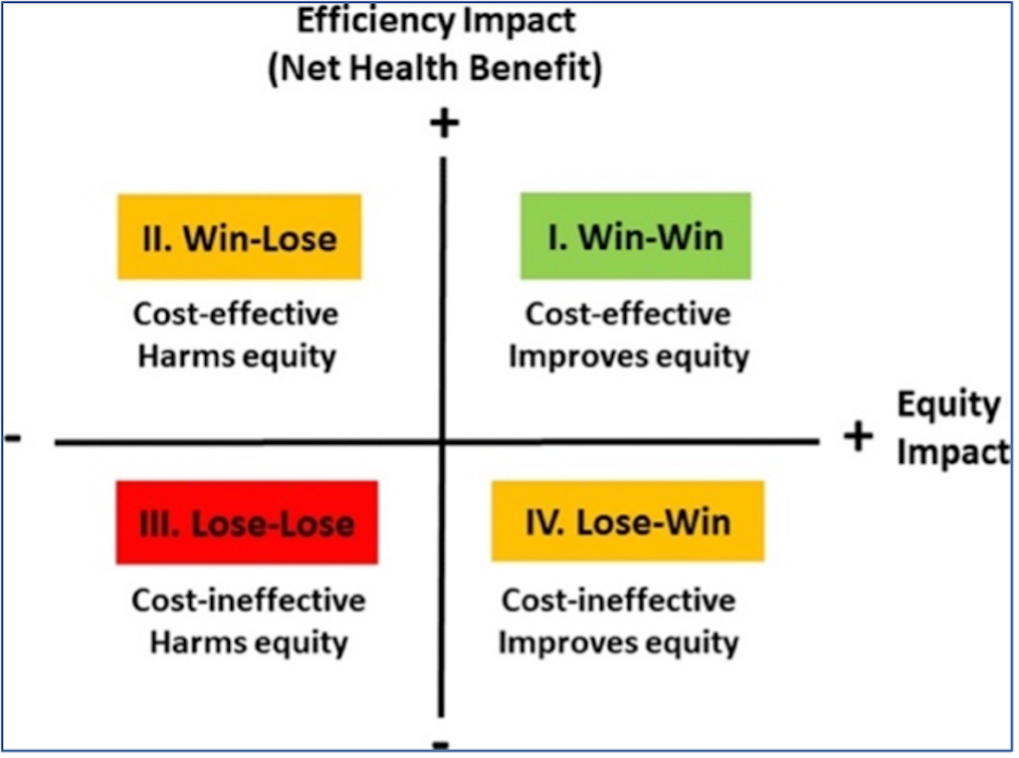
Equity-efficiency impact plane.

We need to rank strategies to fully specify an underlying social welfare function. We will specify the nature of inequality aversion and the level (or value) of inequality. The inequality aversion parameters in these functions describe the trade-off between total health and health inequality (ie, the amount of total health that a decision maker would be willing to sacrifice to achieve an equal distribution). As inequality aversion parameters are difficult to interpret on a raw scale, we will use a more intuitive scale by combining a specific parameter value with a specific health distribution to derive the equally distributed equivalent (EDE) level of health. The difference between the mean level of health in that distribution and the EDE level of health represents the average amount of health per person that one would be willing to sacrifice to achieve full equality in health, given that specific value of inequality aversion. We will use two social welfare indices closely linked to the dominance rules applied above: the Atkinson index [27] to evaluate the distributions in terms of relative inequality and the Kolm index [29] to evaluate the distributions in terms of absolute inequality to estimate the EDE level of health for all strategies. We will visualize our findings using the equity-efficiency impact plane (Figure 4) [30].

##### Sensitivity Analysis

We may run several sensitivity analyses to explore the impact of making alternative assumptions in our modeling on the choice of preferred strategy. We can explore (1) the impacts of alternative assumptions around the distribution of opportunity costs and (2) the impacts of alternative social value judgments about which inequalities are considered unfair.

### Ethical Considerations

The study is under review (24-6035.0) by the University Health Network (UHN) Research Ethics Board (REB). Please note that in accordance with the UHN REB will report any protocol modifications (eg, changes to eligibility criteria, outcomes, analyses) to relevant parties (eg, investigators, REB, trial participants journals, and regulators). As indicated in the earlier section of the manuscript, before joining the Delphi panel, each participant will be approached by a qualitative researcher and study lead to sign an informed consent. Data in the Delphi study will be deidentified and individuals will be identified using a unique study ID. In the compensation process of patient partners, we follow the regulations and protocols of DAC.

## Results

We have submitted the REB application to UHN REB and are expecting to begin recruitment for the Delphi study in the spring of 2025. We anticipate beginning work on the model in the summer of 2025 and completing it by early 2026.

## Discussion

The Delphi study will offer insights into which social factors are perceived as necessary by the stakeholders for guiding the inequity in care access. We may input all social factors into the DCEA model or create distinctive context-dependent scenarios. The findings of this study will provide relevant information on the distribution of the patient population eligible for DR screening, the net health benefit of the intervention, and the health equity impact of DR screening. Applying the DCEA approach will reveal key social factors and drivers that undermine health equity. We anticipate that each of these drivers will raise important questions regarding the implications for decision-making that may have not yet been addressed by Canadian HTA bodies, such as the Canada Drug Agency. We will compare our findings with the relevant literature focused on DCEA.

Distributionally sensitive economic evaluations are expected to result in a more comprehensive valuation of interventions, which is informative to policy makers and governments whose goal is to mitigate health inequities. In our analysis, we will not only assume that all eligible patients receive treatment. This assumption may not hold true in clinical practice, as uptake varying across income quintiles has been observed across many interventions (eg, cancer screening and diabetes complications) [31]. Instead, we will create diverse scenarios where the screening uptake will vary from 25% to 100%. We will apply the “staircase of inequality,” which is a framework used in DCEA to identify the stages of a health program where inequalities may result in costs and health effects varying between equity-relevant subgroups [32].

Our study has several limitations. The study is an illustrative example of applying a DCEA approach based on data available in published literature. Hence, the analysis may only offer a ballpark estimate of the size and direction of the impact on equity and societal welfare, which could be larger or smaller or in the other direction depending on the actual data.

We know that DCEAs are data intensive. This may result in relying on assumptions or a tendency to ignore gradients in model inputs to address data gaps, potentially resulting in uncertainty in the estimates. The introduction of additional analyses as standard practice into the evidence requirements of HTA bodies may increase the burden for those submitting and those appraising the evidence.

### Conclusion

This protocol outlines a study designed to provide information on the inequality impacts of tele-retina screening programs in at-risk patient populations. Understanding the equity impact of the tele-retina program will inform decision makers and program administrators of what may be necessary for creating a contextually and culturally acceptable screening program in line with social context, with the ultimate purpose of improving the screening rate for DR and thus decreasing the incidence of severe vision loss for the at-risk populations. This will be the first study in collaboration with patient partners to mainstream how we assess the economic impact of screening programs and interventions with respect to inequality and access to care for women, people from lower socioeconomic groups, or people from certain cultures or racial backgrounds while remaining focused on economic analysis and the best use of care resources. The study has substantial external validity as the social construct-guided economic model (DCEA) can be applied across disciplines and domains and a range of care settings when evaluating large-scale public health programs that have an explicit goal of tackling health inequality. The “de-novo” integration of social constructs into economic models may be transferred to other screening programs (breast, colon, etc), care settings (primary, secondary, and tertiary), and models of care. We plan to expand this approach to different stroke initiatives.

## Appendix

Multimedia Appendix 1 Social construct variables to be presented to Delphi participants and relative and absolute indices.

## Abbreviations

CDA: Canada Drug Agency
DAC: Diabetes Action Canada
DCEA: distributional cost-effectiveness analysis
DR: diabetic retinopathy
EDE: equally distributed equivalent
HTA: health technology assessment
OHIP: Ontario Health Insurance Plan
REB: Research Ethics Board
SOC: standard of care
UHN: University Health Network

## Data Availability

Data will be presented in the main manuscript.
