# Supplementary material for: Diabetic Retinopathy Screening Among at Risk Populations: Protocol for Distributional Cost-Effectiveness Analysis
Source: JMIR Res Protoc. 2025 Apr 30;14:e60488. doi: 10.2196/60488 (PMC12079062; doi:10.2196/60488)
Supplement: Multimedia Appendix 1 [file resprot_v14i1e60488_app1.docx]

Multimedia Appendix

**Table S1.** Social construct variables to be presented to Delphi participants.

| List of social constructs and variables | Not at all important | Low importance | Neutral | Moderately important | Very important |
| --- | --- | --- | --- | --- | --- |
| Area-level deprivation |  |  |  |  |  |
| Income |  |  |  |  |  |
| Education |  |  |  |  |  |
| Employment |  |  |  |  |  |
| Housing quality |  |  |  |  |  |
| Ethnicity |  |  |  |  |  |
| Area-level ethnic diversity |  |  |  |  |  |
| Sex |  |  |  |  |  |
| Gender |  |  |  |  |  |
| Other (suggest) |  |  |  |  |  |

**Table S2.** Relative and absolute indices.

| Relative inequality indices | Tele-retina screening | Standard of care screening |
| --- | --- | --- |
| Relative gap index (ratio) |  |  |
| Relative index of inequality |  |  |
| Gini index |  |  |
| Atkinson index (ϵ=1) |  |  |
| Atkinson index (ϵ=7) |  |  |
| Atkinson index (ϵ=30) |  |  |
| Absolute inequality indices |  |  |
| Absolute gap index (range) |  |  |
| Slope index of inequality |  |  |
| Kolm index (α=0.025) |  |  |
| Kolm index (α=0.1) |  |  |
